# Comparison of flexible ureteroscopy in the treatment of 1–2 cm single nephrolithiasis and multiple nephrolithiasis

**DOI:** 10.3389/fsurg.2023.1114206

**Published:** 2023-01-30

**Authors:** Guangda Lv, Yongheng Zhou, Wenqiang Qi, Minglei Zhong, Rongyang Li, Yaofeng Zhu

**Affiliations:** ^1^ Department of Urology, Qilu Hospital of Shandong University, Jinan, China; ^2^ Department of Thoracic Surgery, Qilu Hospital of Shandong University, Jinan, China

**Keywords:** flexible ureteroscopy, propensity score matching, retrospective study, solitary nephrolithiasis, multiple nephrolithiasis

## Abstract

**Objective:**

To compare the efficacy of flexible ureteroscopy for single urinary stones with that of multiple urinary stones.

**Methods:**

A retrospective study was conducted on patients who underwent flexible ureteroscopy in Qilu Hospital of Shandong University from January 2016 to March 2021. Propensity score matching was used to match patients with no statistical difference in preoperative clinical data, and they were divided into solitary calculi and multiple calculi two groups. The postoperative hospital days, operation time, complications and stone free rate were compared between the two groups. And multiple stones were divided into high group (S-ReSc > 4) and non-high group (S-ReSc ≤ 4) for analysis.

**Results:**

313 patients were counted. After propensity score matching, 198 patients were finally included in the study. There were 99 cases in the solitary stone group and the multiple stone group. There were no significant differences in postoperative hospital days, complications and stone free rate between the two groups. The operation time of patients with solitary stone group was significantly shorter than that of patients with multiple stones (65.00 min, 45.00 min VS 90.00 min, 50.00 min, *P* < 0.001). The SFR of high group in the multiple stones group was significantly lower than that in the non-high group (7, 58.3% VS 78, 89.7%, *P* = 0.013).

**Conclusion:**

Despite the longer operation time, flexible ureteroscopy has similar outcomes in the treatment of multiple (S-Rec ≤ 4) compared to solitary calculi. Although, this doesn't apply when S-ReSc > 4.

## Introduction

The results of several studies have shown that the incidence of urolithiasis has increased rapidly worldwide in the past 30 years (1, 2). Incidence rates vary from 1% to 19% by region and ethnicity (3, 4). It brings a high burden to social medical resources (5). With the development of intracavitary lithotripsy equipment, the treatment of urolithiasis is also changing. Minimally invasive surgeries such as extracorporeal shock wave lithotripsy, ureteroscopy lithotripsy, and percutaneous nephrolithotomy (PNL) have replaced traditional open lithotripsy and become the main treatment for urolithiasis due to their low complications and excellent lithotripsy effect (6). Especially in ureteroscopy lithotripsy, with the development of equipment, ureteroscope has developed from the initial rigid endoscope to the semi-rigid endoscope and now the flexible endoscope. Its surgical indications have evolved from initial lower ureteral calculi to treat calculi anywhere in the kidney (7). The EAU guidelines state that for 1–2 CM kidney stones, flexible ureteroscopy (f-URS) can achieve similar stone free rate (SFR) as PNL with fewer complications (8). However, the existing guidelines only focus on single calculi, and there is a lack of research on the surgical effect of multiple calculi. Similarly, few studies have explored the surgical effect of multiple kidney calculi (9). This study retrospectively analyzed the safety and efficacy of f-URS in the treatment of solitary kidney stones and multiple kidney stones, and explored whether f-URS has similar effects in the treatment of multiple kidney stones as in the treatment of solitary kidney stones.

## Materials and methods

### Study population and design

This study retrospectively collected patients who received f-URS in Qilu Hospital of Shandong University from January 2016 to March 2021 for the treatment of 1–2 CM kidney stones combined with or without ureteral stones. According to the number of stones, we divided the patients into solitary stone group and multiple stones group. All patients were diagnosed by computed tomography (CT). Preoperative routine blood, urine, urine culture, blood biochemical, liver and kidney function tests, kidney, ureters, and bladder (KUB) examination were performed, and antibiotics were used preventively in all patients during the perioperative period. Patients with positive urine cultures were given sensitive antibiotics based on the culture results. Patients without preoperative CT results should routinely undergo CT examination to determine the number, location, size and other characteristics of stones. Pretreatment insertion of a ureteral stent is required only when a ureteroscope and/or ureteral access sheath (UAS) could not previously be placed through a narrow intramural ureter. In such cases requiring preliminary stenting, RIRS would be carried out after 1–3 week. Inclusion criteria: 1. Patients who received f-URS for kidney stones; 2. The maximum length of kidney stones was between 1 and 2 CM. Exclusion criteria: 1.Solitary ureter stone; 2. Minor patients less than 18 years old; 3. Removal of stones from both sides in one operation; 4. Surgery for other diseases during hospitalization; 5. Surgery for other diseases during surgery; 6. Abnormal renal structure such as solitary kidney, Patients with horseshoe kidney, polycystic kidney, and transplanted kidney; 7. staghorn stones.

We collected preoperative clinical data such as gender, age, American society of Aneshesiologists classification system (ASA), stone burden, stone length, Seoul national university renal stone complexity scoring system (S-ReSc score), pre-stenting numbers, preoperative urine culture, and low calyx stones, as well as postoperative hospitalization days, operation time, complications, stone free rate and other postoperative clinical data. The baseline data of patients were statistically different, so propensity score matching (PSM) was used, and the matching conditions were gender, age, ASA classification, stone burden, stone length, preoperative urine culture, and low calyx stones. Baseline data of matched patients were not statistically different and were comparable. We divided multiple stones into high group and non-high group according to the S-ReSc score, and analyzed whether the number of the renal pelvis and calyces occupied by stones would affect the surgical outcome.

We used the ASA classification to assess the patient's preoperative physical status. Stone length is the length of the largest stone, and if the patient has multiple stones, the stone length is defined as the longest diameter of the largest stone, not the sum of the lengths of all stones taken by other studies. Because we wanted to explore whether single and multiple stones with the same maximum stone length regardless of stone number or stone volume had similar surgical outcomes. Stone burden was calculated using the following formula to calculate the maximum cross-section of the stone: stone length * stone width * 3.14 * 0.25 (10). Therefore, the stone burden is also the largest cross-sectional area of a stone. We used the S-ReSc score to assess the complexity of multiple kidney stones, S-ReSc score was validated for the prediction of SFR after PNL. The score was calculated by counting the number of sites involved, regardless of the size and number of stones (11). The operation time was defined as the time from the insertion of the rigid ureteroscope to the end of the operation. Complications were graded using the Clavien-Dindo grading system (12), and if two grades of complications were combined at the same time, it was defined as the highest grade. SFR was assessed 3 months after surgery using KUB. Completely clear stones or residual stones ≤4 mm were defined as achieving the SFR criteria.

### Surgical procedure

After successful anesthesia, the patient was placed in a lithotomy position and routinely disinfected and sterile surgical sheets were placed. The 8/9.8 Fr ureteroscope was inserted into the bladder through the urethra and the ureteral opening was sought. Then a zebra guidewire was placed in the ureter and the ureteral rigid scope was entered under the guidance of the zebra guidewire, the zebra guidewire was placed up to the renal mons and the ureteroscope was withdrawn. The flexible ureteroscope sheath was passed along the zebra wire into the ureteropelvic junction. A flexible ureteroscope was passed along the ureteral sheath into the ureteral pelvis. After the stone was found, a 200 um holmium laser fiber was inserted and the power was adjusted to 0.6–1.0 J at a frequency of 10–20 Hz to gradually crush the stone. After checking for large stones, a zebra guidewire was placed into the renal pelvis. The flexible ureteroscope and sheath were withdrawn. The ureteroscope was re-entered from outside the zebra guidewire, and a 6F double “J” tube was left in place along the zebra guidewire, and the mirror was withdrawn and the 16 F ureter was left in place.16 FAnd the double “J” stent would be removed 1 month after surgery if no ureteral injuries occurred in procedures.

### Statistical analysis

PSM was performed using RStudio (Version 1.4.1106), indicators included gender, age, stone burden, stone length, ASA, preoperative urine culture, low calyx stones, caliper value = 0.05, radio = 1. Data were statistically analyzed using IBM SPSS Statistics Version 25 (International Business Machines Corporation, Armonk, New York, NY, USA). Shapiro-Wilk-Test was used to identify whether continuous variables conform to a normal distribution or not. If continuous variables do not conform to a normal distribution, use the median and interquartile range to express, and if they conform to a normal distribution, use the mean and standard deviation to express, and the Wilcoxon–Mann–Whitney-Test was used to analyze the differences of binary variables. Dichotomous variables were expressed as percentages, and differences were analyzed using the chi-square test. *P* < 0.05 means there is a statistical difference.

## Results

We counted 313 patients, all of whom successfully completed the operation. The baseline data of the patients were statistically different, so the PSM was used. Finally, 198 patients were included in the study. There were 99 patients in each group. There were no statistically significant differences in baseline clinical data between the two groups. A total of 9 patients in the two groups developed systemic inflammatory response syndrome (SIRS) after surgery, including 4 in the solitary stone group and 5 in the multiple stones group; 3 patients developed postoperative fever, including 1 case in the solitary stone group and 2 cases in the multiple stones group; the above complications are classified as grade 1. There were 3 patients with postoperative sepsis, all of whom were in the multiple calculus group; 1 patient in the solitary stone group with postoperative sepsis complicated with hematuria, which recovered without surgical intervention, the above complications were classified as grade 2. One patient with postoperative pulmonary embolism in the pre-PSM single stone group improved after being transferred to ICU for treatment, and this patient was classified as grade 4; no serious complications above grade 3 after PSM were included in the study. Two patients in the multiple stone group underwent a second session, and none in the single stone group underwent a second operation. The postoperative hospital days, complications, SFR of the two groups were not statistically different, and the operation time in the single stone group was significantly shorter than that in the multiple stone group (65.00 min, 45.00 min VS 90.00 min, 50.00 min, *P* < 0.001) (Table 1).

**Table 1:** Comparison of the clinical characteristics of included patients.

	Before PSM (*n* = 313)			After PSM (*n *= 198)		
	Solitary stone (*n* = 145)	Multiple stones (*n* = 168)	*P*	Solitary stone (*n* = 99)	Multiple stones (*n* = 99)	*P*
Sex (*n*, %)			0.282			0.881
Male	104 (71.7%)	111 (68.7%)		65 (65.7%)	66 (66.7%)	
Female	41 (28.3%)	57 (33.9%)		34 (34.3%)	33 (33.3%)	
Age (year, M, IQR)	50.00, 17	51.00, 16	0.645	48.96 ± 13.09	47.40 ± 12.45	0.393
ASA score			0.072			0.961
I	28	18		14	12	
II	115	150		83	87	
III	2	0		2	0	
Pre-stenting (*n*, %)	3(2.1%)	9(5.4%)	0.131	3(3.0%)	8(8.1%)	0.121
Stone burden (cm^2^, M, IQR)	1.18, 1.57	1.18, 0.98	0.072	1.18, 0.98	1.18, 1.57	0.281
Stone length (cm, M, IQR)	1.50, 0.80	1.45, 0.78	0.001	1.50, 0.50	1.50, 1.00	0.586
Stone destiny (HU, M, IQR)	1102.69, 524.56	1115.03, 504.93	0.201	1128.00, 504.05	1106.39, 476.88	0.192
Urine culture (*n*, %)	9(6.2%)	19(11.3%)	0.164	9(9.1%)	8(8.1%)	0.800
Low pole stones	42	93	*P* < 0.001	40 (40.4%)	41 (41.1%)	0.885
S-ReSc score						
Low (1–2)	145	59		99	34	
Intermediate (3–4)	0	87		0	53	
High (5–9)	0	22		0	12	
Post operation days (day, M, IQR)	3, 2	3, 2	0.225	3.00, 1.00	3.00, 2.00	0.663
Operation time (min, M, IQR)	70.00, 35.00	90.00, 50.00	*P* < 0.001	65.00, 45.00	90.00, 50.00	*P* < 0.001
Complication (*n*, %)	8(5.5%)	15(8.9%)	0.249	6(6.1%)	10(10.1%)	0.297
I	6	10		5	7	
II	2	4		1	3	
III	0	0		0	0	
IV	0	1		0	0	
SFR (*n*, %)	129(89.0%)	150(89.8%)	0.807	91(91.9%)	85(85.9%)	0.175

S-ReSC, Seoul national university renal stone complexity scoring system; M, median; IQR, Interquartile range; PSM, propensity score matching; ASA, American society of Anesthesiologists classification system; SFR, stone free rate.

When multiple stones were divided into high and non-high groups, we found that the SFR was significantly lower in the high group than in the non-high group (7, 58.3% VS 78, 89.7%, *P* = 0.013), with no significant difference in other outcomes (Table 2).

**Table 2:** Comparison of non-high group VS high group for multiple calculi.

	Non-high (S-ReSc ≤ 4) *n* = 87	High (S-ReSc > 4) *n* = 12	*P*
SFR (*n*, %)	78 (89.7%)	7 (58.3%)	0.013
Complication (*n*, %)	9 (10.3%)	1 (8.3%)	1.000
Operation time (M, IQR)	90.00, 50.00	100.00, 57.00	0.334
Post operation days (M, IQR)	3.00, 2.00	3.00, 1.00	0.768

S-ReSC, Seoul national university renal stone complexity scoring system; M, median; IQR, Interquartile range; SFR, stone free rate.

## Discussion

F-URS is increasingly favored by physicians and patients as it has advantage of being minimally invasive and can reach the surgical site through the body's natural channels, making it less damaging to the kidneys and blood vessels (13, 14). The EAU and AUA guidelines state that f-URS can achieve a similar SFR to PNL for kidney stones 1–2 cm in length with lower complications. Evidence for optimal management of multiple ipsilateral stones is limited, and EAU and AUA guidelines do not provide clear recommendations (8, 15). In this study, by comparing the effects of f-URS in the treatment of solitary stones and multiple stones, we explored whether multiple stones would affect the choice of f-URS as the best treatment for 1–2 CM stones.

Our study found that the operative time in the multiple stones group was significantly longer than that in the solitary stone group, which seems logical: more stones obviously require more time. DIOMIDIS et al. showed that the operative time for RIRS was 57.5 min (42 min–90 min) for solitary stone and 88 min (55 min–105 min) for multiple stones, which is consistent with our study (16). However, in their study, semi-rigid ureteroscopy was used to treat solitary calculi, which were all ureteral calculi. In the multiple calculi group, most of them were renal calculi, and f-URS was used for treatment, and UAS was not uniformly used. Therefore, there may be more confounding bias. Although the operative time is generally considered to be no more than 1 h, most of the early studies reported that the operative time of RIRS varied from 60 to 120 min (17). Hua Zhang et al. found that operative time was associated with postoperative fever, and larger stones would lead to longer operative times (18), since longer operative time would lead to longer endotoxin exposure, which greatly increased postoperative fever and the probability of infection. In this study, while controlling for the variable stone length, we found that a higher number of stones also resulted in a longer operative time. Multiple stones, like larger stones, tend to be more likely to be infected stones (19). In order to avoid potential dangers, if the operation time is too long, the operation can be suspended and a second operation can be performed as an option (20), although no safe operation time has yet been determined.

Some studies have found that stone density will prolong the operation time (21), but the threshold of Hu value varies. Mitsuo Ofude et al. found that 863 Hu could be used as the threshold (22), while Matan Mekayten et al. found that for the Standard 20 W Laser, the operation time was significantly prolonged after 1,164 Hu. For the new laser lithotripsy device such as Laser p120w, the operation time is not affected by the Hu value (23). It appears that greater stone density affects postoperative complications and SFR by affecting operative time. However, some studies have found that compared with stone density, stone length is an independent risk factor for predicting postoperative complications (24). Similar results were reported by Kozyrakis D et al., who found that stone density was not an independent risk factor for postoperative complications or SFR, but stone length was (16). In addition, the holmium laser has been the gold standard for laser lithotripsy for decades, and any type of stone can be treated.

Huang et al. reported that they achieved an SFR of 85.7% after a second f-URS (25), which was similar to the multiple stones group in our study. In another study by Breda et al., the final SFR of f-URS in the treatment of multiple kidney stones was 92.2%, which was higher than our study (26). It may be related to that the stones in Breda et al.'s study were all smaller than 1.5 CM, which was smaller than the stone length in our study. Many studies are keen to analyze the independent risk factors affecting postoperative SFR, but the results of each study are controversial. Low pole stones are generally believed to affect the SFR of f-URS, because the anatomical structure of the low renal calyx easily affects the entry of f-URS (27). Studies have found that low calyx stones are not an independent risk factor for SFR, but risk factors for complications. In addition, it was concluded that stone length was an independent risk factor for SFR rather than stone number and location (16), which is similar to our findings. There was no significant difference in SFR between the single stone group and the multiple stone group in our study. However, when we focus on the number of pelvis and calyces occupied by stones, which is how the S-ReSc score works, we found that the effect of f-URS was not good for high stones.

This study is a retrospective study, we use ASA classification to describe the preoperative status of patients, rather than body mass index (BMI), so the results may be biased. And staghorn stones were not included in this study. We did not calculate stone volume, instead, the usage of calculated stone burden was performed. In the use of PSM, the number of included patients is inevitably lost, which may result in patients with important outcome variables such as specific comorbidities ultimately not being included in the study. The small number of patients in the high group may have biased the results.

## Conclusion

Despite the longer operation time, flexible ureteroscopy has similar outcomes in the treatment of multiple (S-Rec ≤ 4) compared to solitary calculi. Although, this doesn't apply when S-ReSc >.

## Data Availability

The raw data supporting the conclusions of this article will be made available by the authors, without undue reservation.
